# Application of Combined Long Amplicon Sequencing (CoLAS) for Genetic Analysis of Neurofibromatosis Type 1: A Pilot Study

**DOI:** 10.3390/cimb43020057

**Published:** 2021-07-23

**Authors:** Sumihito Togi, Hiroki Ura, Yo Niida

**Affiliations:** 1 Center for Clinical Genomics, Kanazawa Medical University Hospital, Uchinada, Ishikawa 920-0923, Japan; togi@kanazawa-med.ac.jp (S.T.); h-ura@kanazawa-med.ac.jp (H.U.); 2 Division of Genomic Medicine, Department of Advanced Medicine, Medical Research Institute, Kanazawa Medical University, Uchinada, Ishikawa 920-0923, Japan

**Keywords:** neurofibromatosis type 1, genetic testing, next-generation sequencing, long-range PCR, multiplex PCR

## Abstract

Elaborate analyses of the status of gene mutations in neurofibromatosis type 1 (NF1) are still difficult nowadays due to the large gene sizes, broad mutation spectrum, and the various effects of mutations on mRNA splicing. These problems cannot be solved simply by sequencing the entire coding region using next-generation sequencing (NGS). We recently developed a new strategy, named combined long amplicon sequencing (CoLAS), which is a method for simultaneously analysing the whole genomic DNA region and, also, the full-length cDNA of the disease-causative gene with long-range PCR-based NGS. In this study, CoLAS was specifically arranged for NF1 genetic analysis, then applied to 20 patients (five previously reported and 15 newly recruited patients, including suspicious cases) for optimising the method and to verify its efficacy and benefits. Among new cases, CoLAS detected not only 10 mutations, including three unreported mutations and one mosaic mutation, but also various splicing abnormalities and allelic expression ratios quantitatively. In addition, heterozygous mapping by polymorphisms, including introns, showed copy number monitoring of the entire *NF1* gene region was possible in the majority of patients tested. Moreover, it was shown that, when a chromosomal level microdeletion was suspected from heterozygous mapping, it could be detected directly by breakpoint-specific long PCR. In conclusion, CoLAS not simply detect the causative mutation but accurately elucidated the entire structure of the *NF1* gene, its mRNA expression, and also the splicing status, which reinforces its high usefulness in the gene analysis of *NF1*.

## 1. Introduction

Neurofibromatosis type I (NF1) is one of the most common autosomal-dominant disorders, occurring with an incidence of one in 2500–3000 individuals, independent of ethnicity, race, and gender [1]. Half of the affected individuals have a de novo *NF1* mutation, while the other half carry a mutation that appears as a familial trait [2]. The clinical features of NF1 are characterised by multiple café au lait macules, Lisch nodules in the iris, and fibromatous tumours of the skin. Less common but potentially more serious manifestations, include plexiform neurofibromas, malignant peripheral nerve sheath tumours, optical pathway and other central nervous system gliomas, scoliosis, tibial dysplasia, and vasculopathy [3]. The diagnostic criteria of NF1, according to the National Institutes of Health Consensus Conference in 1988, are generally accepted worldwide for the current routine clinical practice [4]. The causative gene of the disease, i.e., *NF1*, was first identified in 1990 by Wallace et al. [5]. *NF1* is located on chromosome 17q11.2, spans approximately 290 kb of genomic DNA, contains 58 exons, and encodes a 220–250-kDa cytoplasmic protein called neurofibromin [2]. The protein neurofibromin’s main role is to be a negative regulator of the *RAS* proto-oncogene. Neurofibromin acts as a guanosine triphosphatase (GTPase)-activating protein (GAP), maintaining the proto-oncogene RAS in the inactive GDP form by accelerating the conversion of GTP-RAS to GDP-RAS through the NF1 GAP-related domain (NF1-GRD). The subjects with the disorder have an increased susceptibility to the development of benign and malignant tumours, because RAS is overactivated as a result of the *NF1* loss-of-function mutation [2,3]. Legius syndrome is important for the differential diagnosis of NF1 [6]. This autosomal-dominant inherited disease caused by *SPRED1* mutation is indistinguishable from NF1, since it also produces café au lait macules and axillary or inguinal freckling but does not produce neurofibromas or malignant tumours in contrast to NF1. Café au lait macules are often the only symptom in young patients with NF1, and a distinction is required between these two syndromes for prognostic estimations. The only reliable method for differential diagnosis is genetic testing. *SPRED1* located on chromosome 15q14 belongs to the RAS-MAPK pathway and is involved in the inactivation of RAS together with neurofibromin [7].

Although the causative gene was identified more than 30 years ago, the gene analysis of *NF1* still remains a challenge for several reasons. First of all, *NF1* is a large gene that lacks mutation hotspots, screening all 58 exons being required for merely detecting mutations in the coding region. In addition, aberrant splicing due to deep intron mutations [8] and intragenic- [9] or chromosomal-level large deletions [10] have also been reported. Furthermore, a significant population of patients have mosaic mutations [11]. Additionally, 10 or more *NF1* pseudogenes are present in the human genome, and these highly similar DNA sequences prevent accurate *NF1* mutation analyses [12]. The combination of multiplex ligation-dependent probe amplification (MLPA) and/or other methods of screening for large deletions and RNA- or DNA-based Sanger sequencing achieved very high *NF1* mutation detection rates: 93% and 97%, respectively, in the large cohorts of Korea [9] and France [13]. However, multistep screening and large amounts of Sanger sequencing require labour and time. In recent years, with the advent of next-generation sequencing (NGS), mass sequencing has become easier and faster [14,15]. It was also possible to simplify the analytical procedure. Pasmant et al. showed DNA-based targeted NGS by a multiplex PCR (230 amplicons of ~150 bp) approach could detect point mutations and copy number alterations simultaneously [14]. These achievements are noteworthy, but there is still room for improvement in the NGS method currently in use. Sabbagh et al. showed that a significant proportion of *NF1* missense mutations (30%) were deleterious by affecting pre-mRNA splicing [13]. Additionally, recent studies have shown that many mRNAs bearing premature termination codons (PTCs) escape nonsense-mediated mRNA decay (NMD), in addition to the canonical rules as the NMD evasion of PTCs in the last or penultimate exons [16]. Therefore, the true effect of *NF1* missense and protein-truncating mutations, i.e., whether there is a possibility of protein expression, cannot be determined without the simultaneous testing of DNA and RNA. In addition, the diagnosis of large deletion/duplication mutations by altered exon copy numbers in MLPA and NGS does not determine the mutation itself, the breakpoint sequence on the DNA. From the above point of view, there is a need for a method that can detect mutations in a wide range and determine the nature of mutations in more detail in a single experimental system using NGS.

A similar situation has been observed in another neurocutaneous syndrome, namely tuberous sclerosis complex. Recently, we developed multi-modular long-range PCR-based NGS analysis methods and, also, a combination application of them, combined long amplicon sequencing (CoLAS), to solve this problem [17]. In this pilot study, CoLAS was applied to *NF1* gene diagnosis for five previously reported patients with known mutations, three point mutations and two chromosomal-level microdeletions [12], and in the 15 patients newly recruited for this study. Furthermore, we optimised the method and evaluated its accuracy and utility.

## 2. Materials and Methods

### 2.1. Patient and Sample

The patients participating in this study include those clinically diagnosed by NIH criteria [4] (definite cases) or suspected to have NF1 by partial symptoms but do not fulfil the diagnostic criteria (suspicious cases). The first five patients were cases in which we previously reported *NF1* mutations [12], and their samples were used to optimise and validate the accuracy of NF1 CoLAS. The remaining 15 patients were newly recruited for the present study. Written informed consent was obtained from all patients or parents who participated in this study, and the study design was approved by the Ethics Review Board of Kanazawa Medical University (No. G160, 25 August 2020).

### 2.2. Genomic DNA and Total RNA Extraction and Full-Length cDNA Synthesis

In this study, all DNA samples used were extracted from peripheral whole blood using a rapid extraction method [18]. This method is capable of extracting very-high-molecular-weight DNA, being suitable for long-range PCR amplification. The DNA amount and the optical density (OD) A260/280 ratio were measured using a Nanodrop instrument (Thermo Fisher Scientific, Waltham, MA, USA). The total RNA from peripheral blood mononuclear cells (PBMC) was extracted using TRIzol (Thermo Fisher Scientific). RNA concentrations and OD ratios were measured on a Nanodrop instrument, and the RNA integrity number was measured using a TapeStation 4200 and High-Sensitivity RNA ScreenTape (Agilent Technologies, Santa Clara, CA, USA). Full-length double-stranded cDNA was synthesised from 50 ng of total RNA using a SMART-Seq^®^ HT Kit (Takara Bio USA, Mountain View, CA, USA), according to the manufacturer’s standard protocols.

### 2.3. Long-Range PCR and RT-PCR

**Figure 1:**
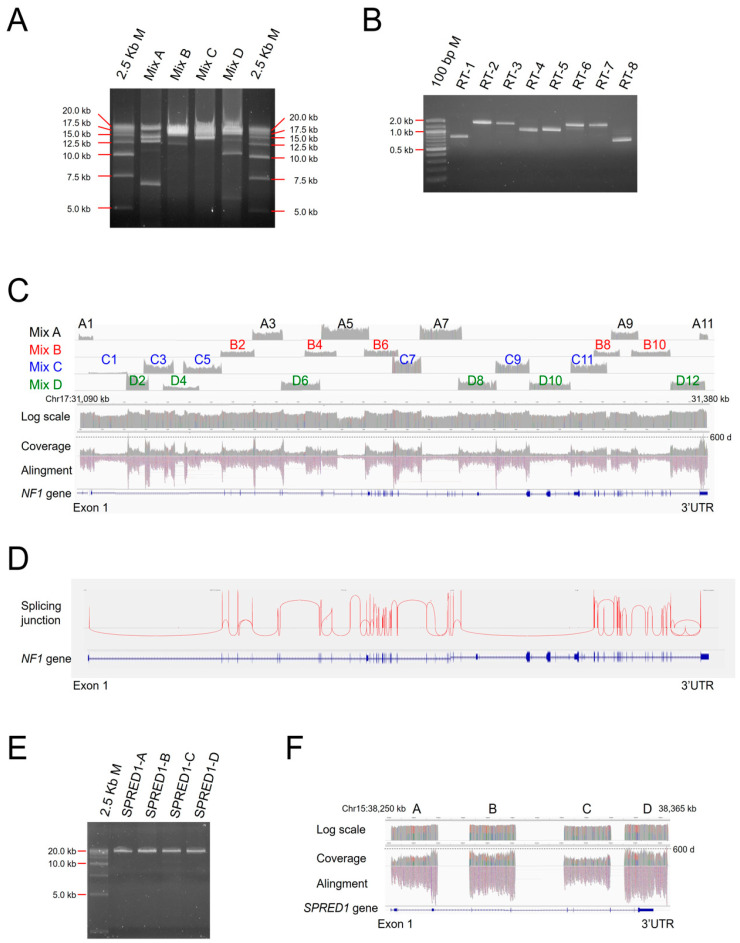
Combined long amplicon sequencing (CoLAS) for NF1 diagnosis. (**A**) The 0.4% agarose gel electrophoresis of *NF1* multiplex long-range PCR products. (**B**) The 1.0% agarose gel electrophoresis of *NF1* RT-PCR products. (**C**) Integrative Genomic Viewer (IGV) bam coverage of *NF1* multiplex long amplicon sequencing (MuLAS). Library preparations were done each A–D multiplex mix amplicon (upper panel) and all pooled amplicons (lower panel). Dashed line in the coverage track indicates a 600 depth. (**D**) Sashimi plot of NF1 reverse-transcribed long amplicon sequencing (rLAS). (**E**) The 0.5% agarose gel electrophoresis of *SPRED1* long-range PCR products. (**F**) IGV bam coverage of *SPRED1* very long amplicon sequencing (vLAS).

We set up several types of long-range PCR-based NGS (long amplicon sequencing; LAS) and arranged them to combine (CoLAS) for the NF1 genetic analysis (see, also, Supplementary Materials). The entire *NF1* genomic region of about 290 kb of chromosome 17 (NC_000017.11:g.31089750_31378015) was amplified by four sets of multiplex long-range PCR for Multiplex LAS (MuLAS) (Figure 1A), and the entire *NF1* mRNA was amplified by RT-PCR with 8 PCR primer sets for reverse-transcribed LAS (rLAS) (Figure 1B).

The long-range PCR primers used in this study were designed by Primer3 v.0.4.0 (http://bioinfo.ut.ee/primer3-0.4.0/ (accessed 30 May 2021)) [19] using the following parameters: primer length, 26–27–30 mer; Tm, 67–67.5–68 °C; Max Tm difference, 0.1 °C; GC%, 45–50–60; and GC Clump, 2, and the other parameters were used with default settings.

To cover the entire genomic region of *NF1*, 23 long PCR primer sets were designed to overlap, with a length from 4102 bp to 21,982 bp. PCR primer sets were divided into four groups (A–D) for multiplex PCR. For RT-PCR, although the SMART-Seq HT kit synthesises full-length mRNA, the *NF1* expression in PBMC is very low, an eight consecutive primer set being required to amplify the entire mRNA. *NF1* type-1 microdeletion break point-specific long-range PCR primers were also prepared according to the literature [20] (Table 1). Moreover, we designed *SPRED1* very long PCR primers, which covered the whole coding exons and surrounding genomic regions.

For the *NF1* multiplex long PCR amplification, each PCR reaction of four multiplex groups contained 1 µL of 20-ng/µL genomic DNA in a 10-µL reaction volume, and two-step PCR cycles were performed with the KOD Multi&Epi enzyme (TOYOBO, Osaka, Japan); after an initial denaturation step at 94 °C for 2 min, there were 35 cycles of 98 °C for 10 s and 68 °C for 10 min, for a total run time of 6 h and 11 min. To obtain uniform amplification products in the multiplex long PCR, the concentration of each primer was experimentally adjusted. The concentrations of the all primers started from 0.1 µM, and the results of the sequence were observed: the concentration of the primer set with a high depth was decreased, and the concentration of the primer set with a low depth was increased. By repeating this step, it was optimized, as shown in Table 1, through 9-, 4-, 3-, and 2-times trials for each of the four groups: A, B, C, and D, respectively.

For the *NF1* RT-PCR, 1 µL of synthesised cDNA was added, and the final concentration of each primer was 0.4 µM in a 10-µL reaction volume, and three-step PCR cycles were performed with the KOD Multi&Epi enzyme; after an initial denaturation step at 94 °C for 2 min, there were 35 cycles of 98 °C for 10 s, 62 °C for 10 s, and 68 °C for 1 min. For the *SPRED1* very long PCR and *NF1* type-1 microdeletion break point-specific PCR, 1 µL was added to 20-ng/µL genomic DNA and a final concentration of 0.15 µM of each primer in a 10-µL reaction volume, and touchdown PCR cycles were performed with the KOD One enzyme (TOYOBO): 3 cycles of 98 °C for 10 s and 74 °C for 10 min, 3 cycles of 98 °C for 10 s and 72 °C for 10 min, 3 cycles of 98 °C for 10 s and 70 °C for 10 min, and 25 cycles of 98 °C for 10 s and 68 °C for 10 min. The total run times were 5 h and 58 min. Although full-length double-stranded cDNA was synthesised from the total RNA of PBMC by a SMART-Seq^®^ HT Kit, it was difficult to amplify the full-length *NF1* cDNA with a single primer set. The maximum length that could be amplified varied according to the sample, and a uniform and stable amplification was obtained finally for all the samples by fractionating them into 8 segments (Figure 1B). It was considered that the low expression level of the *NF1* gene in the blood (low target amount) and the higher-order structure, which long-length cDNA easily take, may affect the amplification efficiency.

### 2.4. Library Preparation and Sequencing

Long PCR products of each sample were pooled and purified using AMPure XP (Beckman Coulter, Brea, CA, USA) by 0.4X, and an NGS library was prepared using a Nextera Flex DNA kit (Illumina, San Diego, CA, USA), according to the manufacturer’s protocols. Libraries were quantified using an HS Qubit dsDNA assay (Thermo Fisher Scientific) and a TapeStation 4200. Qualified size distributions were checked on a TapeStation 4200 using High-Sensitivity D1000 ScreenTape. A 12.5-pM library was sequenced on an Illumina MiSeq system (2 × 250 cycles), according to the standard Illumina protocols (Illumina).

### 2.5. NGS DATA Analysis Pipeline

The FASTQ files were generated using bcl2fastq software (Illumina). The FASTQ files were aligned to the reference human genome (hg38) using the Burrows—Wheeler Aligner MEM algorithm (BWA-MEM version 0.7.17-r1188) [21]. Haplotype variant calling for a single sample was performed using GATK’s HaplotypeCaller (Version 4.0.6.0) [22]. The SNVs and INDELs were functionally annotated by SnpEff (Version 4.3t) in order to classify each variant into a functional class (HIGH, MODERATE, LOW, and MODIFIER) [23]. For variant annotation, the Database of Short Genetic Variations dbSNP (Version 151) and ClinVar were used [24,25]. For targeted RNA sequencing, the paired-end 250-bp reads were aligned to the reference human genome (hg38) using HISAT2 (Version 2.1.0) [26]. For visualisation, IGV (Version 2.4.13) was used [27]. To detect large INDELs, Pindel (Version 0.2.5b9) was used [17,28].

### 2.6. In Silico Analysis of Missense Variants

For missense mutations whose pathological significance has not been determined, an in silico analysis was performed with Variant Annotation Integrator (http://genome.ucsc.edu/cgi-bin/hgVai?hgsid=723950191_wYiZpDqQkfTaYfcMalCmmczL927z, last accessed on 30 May 2021) and Combined Annotation-Dependent Depletion (CADD) (https://cadd.gs.washington.edu/, last accessed on 30 May 2021). Variant Annotation Integrator is a simplified version of dbNSFP [29] that runs on the website, and the results of multiple mutation prediction programmes are output at once. These programs include SIFT (https://sift.bii.a-star.edu.sg/, last accessed on 7 July 2021), PolyPhen-2 (http://genetics.bwh.harvard.edu/pph2/, last accessed on 7 July 2021), Mutation Taster (http://www.mutationtaster.org/, last accessed on 7 July 2021), Mutation Assessor (http://mutationassessor.org/r3/, last accessed on 7 July 2021), and the Likelihood ratio test (http://www.genetics.wustl.edu/jflab/lrt_query.html, last accessed on 7 July 2021). CADD is a framework that integrates multiple annotations into one metric by contrasting the variants that survived natural selection with simulated mutations. The results are expressed as PHRED-like scaled C-scores, and usually, greater than 20 is adopted as the cut-off value. CADD ranking is a variant relative to all possible substitutions of the human genome. A C-score greater than 20 means the variant is ranked within the 1% of the most deleterious variants [30]. In this study, we tentatively defined that a missense variant could be pathogenic when all of the following conditions were met: frequency of the general population in dbSNP was less than 0.001, majority of the programmes of Variant Annotation Integrator determined the variant as pathogenic, and the PHRED-like scaled C-score of CADD was above 20.

### 2.7. Heterozygosity Mapping of NF1 Region

Since MuLAS analyzes the entire *NF1* genomic region, including the intron sequence, it is possible to determine whether the copy number of the region is kept at two by detecting the heterozygous variants. In addition, if there is a large deletion or insertion within the long-range PCR region where heterozygosity is maintained, it should be detected, and the break points were accurately analysed by Pindel software [28]. Notwithstanding, no intragenic large deletions or insertion of the *NF1* were detected in this study.

There are many polymorphisms in introns, but many heterozygous false positives are also detected, because the error call rate of NGS in repetitive DNA sequences is high [17]. To eliminate these false positives, two samples, NF_04 and NF_05, were used with the previously reported large deletions at the chromosomal level [12]. A total of 177 heterozygous variants called by HaplotypeCaller in these samples could be determined to be false positives, because these samples have only one allele of *NF1* and heterozygous variants cannot occur. Most of them were due to repetitive sequences, and some seemed to be sequence-dependent PCR errors. True heterozygous variant candidates were extracted in each sample by removing these false positives from heterozygous calls by HaplotypeCaller. Finally, true heterozygous variants were determined by visually checking with IGV. Since many SNPs are common to multiple samples, the number requiring visual inspection decreased as the number of samples increased. For heterozygosity mapping of the *NF1* region, the number of heterozygous variants in each long-range PCR region was plotted in each patient.

### 2.8. Detection of Chromosomal-Level Large Deletion by Break Point-Specific Long-Range PCR

According to previous studies, two recurrent chromosomal microdeletions are found in patients with NF1 [31]. These microdeletions have specific break points located in paralogous regions flanking *NF1*: proximal *NF1-REP-a* and distal *NF1-REP–c* for the 1.4-Mb type-1 microdeletion and *SUZ12P1* and *SUZ12* for the 1.2-Mb type-2 microdeletion. Type-1 microdeletion is a major type (about 80%), while type-2 microdeletion is relatively rare (about 10%). NF_04 and NF_05 are known to have a type-1 microdeletion, according to a previous study. The proximal break point of the type-1 microdeletion is located between *LRRC37BP1* and *SUZ12P1*, and the distal break point is located on the telomere side of *LRRC37B* [32], and the region containing break points can be amplified by long PCR (Table 1) [20].

### 2.9. Accessing Features of NF1 Splicing Mutations

Since RT-PCR uses the full-length cDNA as a template, although it is amplified by 8 fractions, it shows the overall mutant allele expression and splicing state of *NF1* mRNA. In addition, PCR amplifications of both alleles at the heterozygous points are not always equal [33], but the differences in amplification at the DNA level (MuLAS) can be used to correct the allele expression ratio at the mRNA level (rLAS).

### 2.10. Sanger Sequencing

To validate the NGS results, each *NF1* exon PCR was performed as previously reported [12], and direct DNA sequencing was performed using the BigDye Terminator v3.1 cycle sequencing kit and ABI PRISM 3100xl Genetic analyser (Thermo Fisher Scientific).

### 2.11. Statistical Analysis

The differences of the *NF1* allelic expressions were analysed by unpaired-*t* tests, and *p* < 0.05 (double-tailed) was considered statistically significant.

### 2.12. DATA Deposition

The raw data (fastq files) in this study were deposited in the Japanese Genotype–phenotype Archive (JGA) (https://www.ddbj.nig.ac.jp/jga/index-e.html, last accessed on 30 May 2021), study:JGAS000288 (https://humandbs.biosciencedbc.jp/en/hum0271-v1, last accessed on 30 May 2021).

## 3. Results

### 3.1. Clinical Features of the Patients with Neurofibromatosis Type 1

A total of 20 patients with neurofibromatosis type 1, including suspicious cases, participated in this study. A summary of the clinical features of the included patients in this study are listed in Table 2. Patients NF_06, 09, and 16 were suspected of having NF1 by café au lait macules and central nervous system symptoms, while NF_13 had only multiple café au lait macules. In these patients, other NF1 symptoms were not present, and the patients did not meet the clinical diagnostic criteria. However, these patients may not have cutaneous and/or plexiform neurofibroma due to their young ages.

### 3.2. CoLAS for Genetic Analysis of NF1

Multiplex DNA amplicons of *NF1* were pooled and sequenced by NGS, Multiplex LAS (MuLAS). As a result, uniform coverage was obtained (Figure 1C). Additionally, *NF1* RT-PCR amplicons were pooled and performed a junction sequence analysis by NGS, reverse-transcribed LAS (rLAS), and the splicing state of the full-length *NF1* mRNA was reconstructed (Figure 1D). Four primer sets of very long-range PCR (around 20-kb single amplicons) for the *SPRED1* gene was set up to differential the diagnosis of Legius syndrome (Figure 1E), and NGS sequencing was performed, very LAS (vLAS) (Figure 1F).

As a result of sequencing, all long-range PCR products were amplified almost evenly, and sequence reads were formed seamlessly. Therefore, the coverage rate for the target sequence (defined as the percentage of bases with a depth of 40 or more on the IGV among the bases between each PCR primer) reached 100%. The average depth of MuLAS and vLAS was 200X (at least 40X), and rLAS was 600X (at least 100X). We evaluated the CoLAS running costs at approximately ¥10,000 (Japanese yen) per sample, including DNA and RNA extraction, cDNA synthesis, library preparation, NGS sequencing, and Sanger sequencing.

### 3.3. Point Mutation Detection of NF1

MuLAS detected various point mutations at the DNA level (Table 3). In the three cases previously reported mutations by Sanger sequencing—namely, in NF_01–03—the same mutation was also detected in this study [12]. Of the 15 newly analysed patients, 8 out of 11 clinically definite cases were found to have variants that were thought to be disease-causative mutations. NF_07, 08, 17, and 19 had protein-truncating mutations, and two of them were newly identified mutations in this study. NF_20 had a known start codon mutation [13,34], and NF_10, 11, and 12 had infrequent missense variants and had varying degrees of clinical significance, according to ClinVar (https://www.ncbi.nlm.nih.gov/clinvar/ (accessed on 30 May 2021)). On the other hand, protein-truncating mutations were identified in two out of the four suspicious cases (NF_06 and 16), and one of which was a novel mutation.

An in silico analysis was performed for missense mutations whose pathological significance has not been determined (Table 4). To varying degrees, these mutations were determined to be potentially pathogenic.

**Figure 2:**
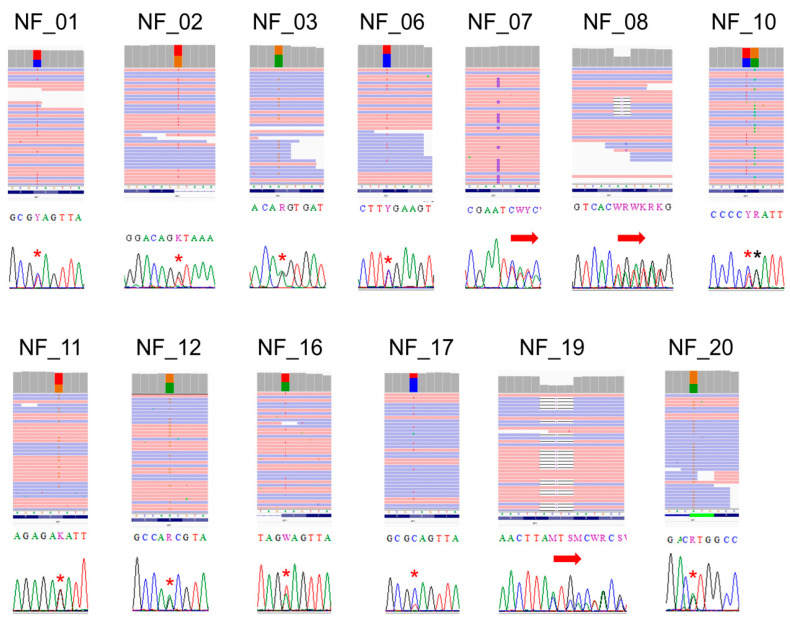
NGS (IGV) and Sanger validation of the detected *NF1* mutations. Single-base substitutions are indicated by stars, and frameshifts are indicated by arrows. Note, in NF_10, NGS, but not Sanger sequencing, shows the mutation, and SNP are located on a different allele. In NF_17, both NGS and Sanger show an unbalanced mutation allele frequency, indicating a mosaic mutation.

These NF1 variants detected by NGS were validated by Sanger sequencing (Figure 2), and all of them were confirmed. In NF_10, the missense variant c.2033C>T p.Pro678Leu was detected with a synonymous variant c.2034G>A p.Pro678=, which is a common SNP (rs2285892) with high allelic frequency in the general population, A = 0.439172 (55146/125568, TOPMED). Sanger sequencing cannot distinguish if these two variants are located on a same allele (in-cis) or different alleles (in-trans); since NGS sequences on a molecule-by-molecule basis, it has been shown that these variants are present in different alleles (in-trans). As every c.2033C>T and c.2034G>A substitution appeared in different reads, no read contained both of them. The nonsense mutation of NF_17, c.625C>T p.Gln209Ter, was considered as mosaic. GATK’s HaplotypeCaller detected a low frequency of mutation allele (T = 0.25), and the Sanger sequence also detected a low T peak.

### 3.4. Heterozygosity Mapping of the NF1 Region

Heterozygous variants were extracted in each sample, and the numbers in each long-range PCR region were counted (Table 5). In 12 samples, the presence of single-nucleotide variants (SNV) or short insertion/deletion variants (Indel) demonstrated heterozygosity of all long-range PCR regions, i.e., the presence of both alleles’ entire *NF1* gene. What should be noted is, since long-range PCR regions next to each other overlap, in the region sandwiched between two regions where maintaining two gene copies has been confirmed by heterozygous variants, the existence of two copies of the gene is guaranteed, even without a heterozygous variant in that region, because both the forward and reverse PCR primers used to amplify that region were located in the adjacent regions that confirmed the existence of two copies. In the remaining samples, there was an area where the copy number could not be determined only from the MuLAS data. In 18 samples, excluding the *NF1* type-1 microdeletion sample (NF_04, 05), the determination rate of heterozygosity was 85.3% in all the primer sets and 86.9% in the primer set containing the *NF1* exons.

### 3.5. Detection of Chromosomal Level Large Deletion by Break Point-Specific Long-Range PCR

**Figure 3:**
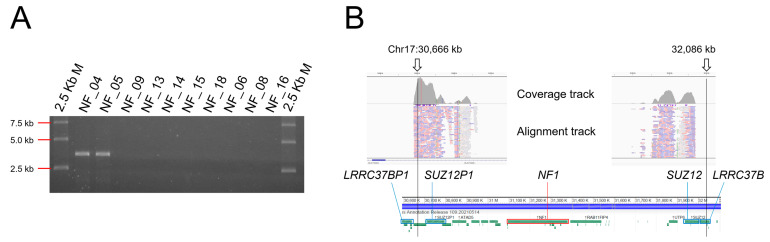
Detection of the type-1 microdeletion of *NF1*. (**A**) The 0.5% agarose gel electrophoresis of type-1 microdeletion-specific long PCR. (**B**) NGS mapping of the PCR amplicon of NF_04. This PCR product was mapped separately at a distance of 1420 Kb containing *NF1* (31,090 kb–31,380 kb) as the expected loci described in the text. On the alignment track, pink and purple colours indicate the direction of the read strands, and white indicates the reads mapped to multiple regions due to pseudogenes.

The break point PCR for type-1 microdeletion was performed, and approximately 4 kb of product was obtained specifically in NF_04 and 05 (Figure 3A). The PCR product was microdeletion-specific and was not found in the other samples. Moreover, we also performed a NGS analysis of this PCR product and mapped it to two regions 1420 kb apart on both sides of *NF1*, as expected (Figure 3B).

### 3.6. Accessing Features of NF1 Splicing Mutations

**Figure 4:**
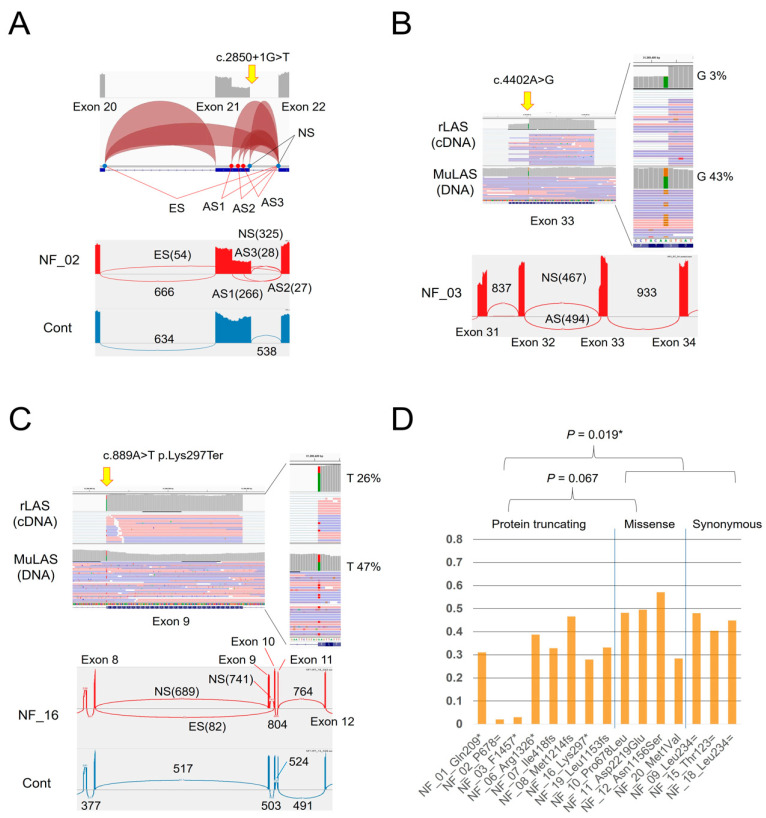
*NF1* splicing analysis by rLAS. (**A**) The NF_02 exon 21 splicing donor site mutation showed three different aberrant splicings and exon 21 skipping. The splice junction track (upper) and sashimi plot (lower) of IGV are shown. (**B**) In NF_03, this similar look to missense mutation actually causes abnormal splicing, creating a new splicing acceptor site. The IGV coverage track, alignment track, and sashimi plot are shown. (**C**) NF_16 missense substitution is the first base of exon 9, and it causes exon 9 skipping in minor populations. (**D**) Mutation allele expression ratio. AS, aberrant splicing; ES, exon skipping; and NS, normal splicing. Numbers in the sashimi plot indicate the junction read numbers.

The analysis using rLAS detected splicing anomalies in three samples. In NF_02, very complex aberrant splicing events caused by the exon 21 splicing donor site mutation NM_000267.3:c.2850+1G>T were observed (Figure 4A). Three different aberrant splicing events into exon 21 using three different GT sequences as new splicing donor sites were detected with various frequencies. The first two were out-of-frame deletions (232 and 144 bp), while the last one was an in-frame deletion (90 bp). The junction read number of each aberrant splicing was 266, 28, and 27, respectively, compared to exons 21 and 22 normal splicing, which was 325. Additionally, exon 21 skipping was observed as junction read number 54. The overall splicing anomaly is described at the RNA level as NM_000267.3: r.[2410_2850del;2619_2850del;2707_2850del;2761_2850del]. Among them, exon 21 skipping and r.2619_2850del were not detected in a previous study that used random primed cDNA and local RT-PCR [12].

As previously reported in NF_03, this seemingly missense mutation, NM_000267.3:c.4402A>G p. (Ser1468Gly), is actually a splice mutation that creates a new splicing acceptor site in exon 33 and the stop codon immediately after that, NM_000267.3:c.4402A>G r.4368_4402del p.Phe1457Ter [12]. In a previous study, it was not possible to quantify the efficiency of this abnormal splicing. Therefore, it was unclear whether the effects of missense amino substitution p.Ser1468Gly would remain or not. In the present study, this quantification was possible by using NGS. Comparing the DNA (MuLAS) and RNA (rLAS) levels, it was shown that mutant bases (G) are contained in only 3.5% of mRNA (corrected by the allele ratio in the DNA level), because the G base is almost spliced out as an acceptor site (Figure 4B, upper part). The sashimi plot also showed that this aberrant splicing junction number was nearly equal to the normal splicing junction number from the wild-type allele (Figure 4B, lower part). Overall, the major effect of this one base substitution was demonstrated to be protein truncating associated with aberrant splicing.

In this study, we also detected another splicing anomaly in the NF_16 nonsense mutation, NM_000267.3:c.889A>T p.Lys297Ter. This A-to-T substitution is located at the first base of exon 9. rLAS demonstrated that the mutant base (T) expression level in mRNA was reduced to 27.7% (corrected by the allele ratio at the DNA level), and there was a minor population of exon 9 skipping (Figure 4C).

Moreover, the mutant allele expression ratio was compared to the wild-type allele at the mRNA level (corrected by the allele ratio at the DNA level) between the protein truncating mutations (frameshift, nonsense, and splicing mutations) and missense mutations. For NF_02, the polymorphism detected in this sample, NM_000267.3:c.8151G>A p.Pro2717=, was used because the mutant base was spliced out and was not included in the mRNA. NF_03 was classified as a protein-truncating mutation based on the above discussion. NF_17 was excluded due to a mosaic mutation. Protein-truncating mutations had the impression that a lower allele expression level than that of the missense mutations and a significant difference was observed (*p*-value = 0.019) when the synonymous polymorphism of the sample for which no mutation was identified was added (Figure 4D). It is suggested that protein-truncating mRNA may be destroyed by nonsense-mediated mRNA decay (NMD). However, this difference of mRNA expression is largely due to the two samples of NF_02 and NF_03, and it should be noted that there are some exceptional samples, such as NF_08, that have a hardly decreased mRNA level despite the frameshift mutation in exon 27. It suggests the sure existence of a NMD evasion case that does not follow the canonical rules. Additionally, NF_20 is a start codon mutation, NM_000267.3:c.1A>G p.Met1Val, being classified as missense mutation, but the mRNA level of the mutant allele may be decreased. However, no splicing abnormalities were observed in rLAS.

In conclusion, the abnormal splicing and allelic expression of *NF1* mRNA could be observed accurately at the same time by combining MuLAS and rLAS data. Therefore, CoLAS is a useful tool that can comprehensively explore the effects of the point mutations on mRNA transcription.

### 3.7. SPRED1 Mutation Analysis

For the differential diagnosis of Legius syndrome, *SPRED1* vLAS was added for the patients with no identified *NF1* mutations (NF_09, 13, 14, 15, and 18) and for the patients with missense variants of undetermined pathological significance (NF_10, 11, and 12). No *SPRED1* mutation was detected in these patients, including point mutation and large deletion/insertion. Additionally, all long amplicons contained heterozygous polymorphisms, confirming that, in all the patients, the entire *SPRED1* gene region was maintained in two copies.

### 3.8. No NF1 Mutation Detected Cases

Patients NF_09 and 13 did not meet the diagnostic criteria for NF1, and their parents did not wish to pursue genetic testing. Regarding NF_14, the heterozygous mapping by MuLAS was incomplete, and MLPA was added at another facility, but no copy number abnormality was detected in the *NF1* exons. CoLAS-denied point mutations, large deletions/insertions, and splicing mutations, including deep intron mutations, leave the possibility of low-frequency mosaic mutations in patients who met the clinical diagnostic criteria but *NF1* mutations could not be identified (NF_14, 15, and 18).

## 4. Discussion

The diagnosis of a typical NF1 patient is easy, based on the clinical diagnostic criteria, but genetic testing has different significance. Frequently, young patients often present only with café au lait macules, and if there is no family history, the diagnosis cannot be confirmed until other symptoms appear. On the other hand, an optic glioma is commonly found in young children with NF1 and sometimes result in visual impairment. Additionally, the frequency of occurrence of optic glioma is known to have a genotype-phenotype correlation that *NF1* mutations located in a third of the 5′ side show a significantly higher risk [35]. Another important point is that the difference of phenotypic expressivity in NF1 is extremely large, forming a wide spectrum from very severe cases to almost asymptomatic mild cases. Patients with mosaic mutations that are present in a non-negligible proportion contribute to one reason for the phenotypic mildness. Therefore, even in adult patients, the diagnosis of atypical cases is dependent on genetic testing. Actually, in this study, definitive *NF1* mutations were detected in two young patients that did not fulfil the clinical diagnostic criteria (NF_06 and NF_16).

Recently, clinical genetic testing has become basically performed using NGS. However, in most cases, it is all about doing massive sequences in the coding and exon/intron boundary region and copy number analyses. There are still insufficient points, such as in genetic testing. As in NF1, the causative gene is not only large, but also, the pattern of gene mutation is diverse. In addition, mutations at the DNA level have various effects on mRNA splicing. Additionally, determining the break point sequences of DNA for large intragenic deletions/duplications is not easy. Therefore, we developed a method that can detect as many types of gene mutations as possible and analyse the details of the nature of that on a single NGS platform. CoLAS, which simultaneously analyzes the entire genomic region and the full-length cDNA of a specific gene based on long-range PCR, has already improved the genetic analysis of the tuberous sclerosis complex [17]. In this pilot study, CoLAS was applied to the genetic diagnosis of NF1 and also demonstrated its high utility.

There are many advantages of using a long-range PCR as a base for a NGS analysis. Pseudogene sequences can be eliminated by designing target-specific PCR primers. An accurate sequence can also be obtained for introns. These are difficult with the capture probe method, and at the same time, it is less expensive to order PCR primers than to make a new capture probe for a specific gene. Long-range PCR products are likely to contain heterozygous bases, allowing a copy number confirmation and detection of the intragenic structural abnormalities (large deletions/insertions) simultaneously. By making it a multiplex, the amount of template DNA can be reduced. In this study, the *NF1* genomic region spanning about 290 kb can be covered by only four PCR reactions (total of 80-ng genomic DNA). By sequencing the full-length cDNA, the splicing state and allele expression of the entire gene can be understood, and the splicing abnormality associated with the deep intron mutation can be detected by comparison with the DNA sequence.

In the present study, *NF1* mutations were identified in 10 of the 15 newly analysed patients. It was possible to quantitatively measure that a single-point mutation causes various splicing abnormalities at the same time and that splicing abnormalities are caused by mutations that appear to be missense or nonsense mutations. The mapping of the *NF1* copy number using heterozygous polymorphism confirmed the presence of two copies in the entire region of the *NF1* gene in 12 out of 18 patients, excluding two cases of type-1 microdeletion, and the determination rate of heterozygosity was 86.9% in the primer set containing *NF1* exons. If no heterozygous polymorphism is found in the *NF1* region, microdeletion is suspected, and it was shown that type-1 microdeletion can be directly proved by break point-specific long-range PCR.

Additionally, this pilot study revealed the current limitations of NF1-CoLAS. First of all, with any type of LAS, it is difficult to accurately measure the alterations of gene copy numbers. Accordingly, we tried heterozygous mapping using intron SNPs, but some patients had regions where heterozygosity could not be proved, and an additional analysis by MLPA was required. The low frequency of polymorphisms in some patients may be due to the effect of recruiting a single ethnic group (Japanese).

Next, the detection of mosaic mutation is incomplete. In patients with no identified *NF1* mutations, the possibility of less-frequent mosaic mutations remains. Increasing the NGS reading depth may detect less-frequent mosaic mutations but, at the same time, increase the risk of detecting false positives associated with PCR errors. Some PCR errors occur in a DNA sequence-dependent manner, and others occur randomly. To avoid false positives, it is necessary to duplicate experiments to extract common mosaic mutations and then remove the known sequence-specific false positives [17]. However, to create a sequence-dependent false-positive list, it is necessary to analyse more samples, which is an issue to consider in the future.

This method could also be useful in analysing somatic mutations in tumours involving the *NF1* gene. In that case, a frozen tumour sample is required to extract high-molecular-weight DNA and RNA. In addition, more accurate mosaic mutation detection is required.

At the end, in rLAS, the mRNA expression pattern of *NF1* was analysed in lymphocytes, but it is expected that the expression of *NF1* has tissue specificity, and how accurately it reflects the aberrant expression in the diseased target organ (nervous system) is unknown.

## 5. Conclusions

Although there is a limit on the sample numbers, this pilot study showed that CoLAS is an excellent method for the precise genetic analysis of NF1. This NGS application can not only simply identify disease-causative mutations (10 out of 15 new patients, including suspicious cases) but also accurately shows the detailed individual genomic structures, including chromosomal-level microdeletion, quantitative mRNA transcription, and the splicing state of the *NF1* gene. Additionally, there are still remaining issues to be noted, such as determining the gene copy numbers, identifying less-frequent mosaic mutations, and tissue-specific gene expression.

## Figures and Tables

**Table 1:** Long PCR primers for NF1 genetic testing.

Primer Set	Primer Name	Primer Seq	Primer Position	Product Size (bp)	Final Primer Conc. µM	Multiplex Group
Multiplex long PCR primers			NC_000017.11 (chr17, GRCh38.p13)			
NF1-A1	NF1_1FN	5′-TTTCATTGTCTTTCTCCAAAGCACAGG-3′	g.31089750_31089776	6776	0.038	A
	NF1_1RN	5′-CCTTAAACCATCCCCAACTACCTACAGC-3′	g.31096498_31096525			
NF1-C1	NF1_In1_1F	5′-TGGCTATGAGTTTTGCAGAGGAAAGC-3′	g.31094323_31094348	17,828	0.5	C
	NF1_In1_1R	5′-TGAGACAAAAACAGCTCAAGGGTTCC-3′	g.31112125_31112150			
NF1-D2	NF1_In1N_1-2F	5′-AAACGCTTAAAAGCAGCCAGAAGAGG-3′	g.31111486_31111511	10,488	0.07	D
	NF1_In1N_1-2R	5′-ACTGTGCTAAGCCTGGGGATATTAATGG-3′	g.31121946_31121973			
NF1-C3	NF1_In1N_2S	5′-TCTGGATATTAGCCCTTGGTCAGATGG-3′	g.31119500_31119526	13,970	0.1	C
	NF1_In1N_2AS	5′-GGGTCTTGAAGTGGTATCTGCACACC-3′	g.31133444_31133469			
NF1-D4	NF1_In1_3F	5′-GCATGGTTGCTTTGTAGTGTCACTGG-3′	g.31128397_31128422	16,667	0.3	D
	NF1_In1_3R	5′-GAACGAAGAGGAAAATCTAGGGGATGC-3′	g.31145037_31145063			
NF1-C5	NF1_In1_4F	5′-TGTGCAGCTATCATCCTGTGTTCTCC-3′	g.31137591_31137616	17,819	0.2	C
	NF1_In1_4R	5′-AGCACCCTCAGCTACACAATTGAAGG-3′	g.31155384_31155409			
NF1-B2	NF1_2-5F	5′-GCCCCCTCCTTTACACTCTAAAAATGC-3′	g.31154673_31154699	15,742	0.042	B
	NF1_2-5R	5′-ATCACAATCTCCTCGTTCCATTCTGC-3′	g.31170389_31170414			
NF1-A3	NF1_5-8F	5′-CCTGCCCTCACCTTGTATATCTCTTGC-3′	g.31169247_31169273	14,116	0.038	A
	NF1_5-8R	5′-CATGAAGGAAACCATCTCATGTTCAGC-3′	g.31183336_31183362			
NF1-D6	NF1_In8_FN	5′-GCCCTTGGGTTTTTACATAGTGTCAGC-3′	g.31182416_31182442	18,077	0.12	D
	NF1_In8_RN	5′-GCACTTTCTGTCAGCTGCCTACTTCC-3′	g.31200467_31200492			
NF1-B4	NF1_9-10F	5′-TGTGATGCACATTGAAGTTTGAGAACC-3′	g.31193253_31193279	14,719	0.04	B
	NF1_9-10R	5′-GAAAATTATGGGCAGCTGGTAAAGTGG-3′	g.31207945_31207971			
NF1-A5	NF1_10-15F	5′-GTATGGGTGCTTTGTGCTTCTTCTGG-3′	g.31200898_31200923	21,982	0.1	A
	NF1_10-15R	5′-ACTCCAGAGCTGCACTGTCTAACATGG-3′	g.31222853_31222879			
NF1-B6	NF1_15-29F	5′-CTCAAAAGGAAAAAGCTGCACACATAGG-3′	g.31220468_31220495	15,765	0.05	B
	NF1_15-29R	5′-TGTCCCTGGATCTAAGGCAAATAAAAGG-3′	g.31236205_31236232			
NF1-C7	NF1_In29_F	5′-GGTGATTTTTCAGCTGTAGGGAAGTGG-3′	g.31233344_31233370	13,515	0.08	C
	NF1_In29_R	5′-AAGAGATTCCGCAGGCAGACTTACTAGG-3′	g.31246831_31246858			
NF1-A7	NF1_30-36F	5′-ACTCAGCACTTCCAGAGATTCCAAGG-3′	g.31245995_31246020	19,438	0.07	A
	NF1_30-36R	5′-AAGTCAACTGGGAAAAACCAAACTTGC-3′	g.31265406_31265432			
NF1-D8	NF1_In35_1F	5′-TTTGACATCACTGAGGACATCCTAGCC-3′	g.31263534_31263560	17,737	0.1	D
	NF1_In35_1R	5′-TGAGAATAGCCCAGAAGTTCACACAGG-3′	g.31281244_31281270			
NF1-C9	NF1_In35_2F	5′-AGGCTGTGGGAAGGATATTGTAAGTGG-3′	g.31280589_31280615	15,711	0.11	C
	NF1_In35_2R	5′-CCTGTTCATCCTTCTGTTTCTCACACC-3′	g.31296273_31296299			
NF1-D10	NF1_In35_3F	5′-TCCAGGGTCCTCAGATTGGTATATTGG-3′	g.31296081_31296107	18,983	0.1	D
	NF1_In35_3R	5′-TACTCAGCACCACTGATGAGAGAAATGC-3′	g.31315036_31315063			
NF1-C11	NF1_In35_4F	5′-ACAGCGTACGAGAGTTCCATTTTCTCC-3′	g.31314920_31314946	16,782	0.11	C
	NF1_In35_4R	5′-CTTATGGTGTACGGTGAAGACGAAGACC-3′	g.31331674_31331701			
NF1-B8	NF1_37-48FN	5′-AGAGCTCCAGATGGGTCATTCCTACC-3′	g.31325474_31325499	12,099	0.02	B
	NF1_37-40R	5′-TCCAACAAAGCTTCTGTGACTGTTTCC-3′	g.31337546_31337572			
NF1-A9	NF1_40-48FN	5′-AGCATCCAGAGGGATATTCTCAGTACCC-3′	g.31333463_31333490	12,591	0.013	A
	NF1_40-48RN	5′-TTGCCACGTCTTTGTACTGTCTTAGCC-3′	g.31346027_31346053			
NF1-B10	NF1_48-57F	5′-ATTCTAGTGCCCTTTGGATGCTAGGC-3′	g.31342682_31342707	18,097	0.09	B
	NF1_48-57R	5′-CTGATCACAAGGGAATTCCTAATGTTGG-3′	g.31360751_31360778			
NF1-D12	NF1_In56_F	5′-AGAGCCAGCTTAGTATCACTGCCAACC-3′	g.31360626_31360652	15,976	0.11	D
	NF1_In56_R	5′-TCTCAAGGGACACATAGTGGTGAGAGG-3′	g.31376575_31376601			
NF1-A11	NF1_58-UTR-F	5′-TCCTAGAATGTGTCCCCGTTGTTAAGC-3′	g.31373914_31373940	4102	0.015	A
	NF1_58-UTR-R	5′-TTCTATAGCGGGAAAGCTGAAAAGTTGG-3′	g.31377988_31378015			
Large genomic deletion (type-1 microdeletion)-specific primers ^#^			NC_000017.11 (chr17, GRCh38.p13)			
NF1-PRS2	NF1-PRS2-F	5′-TCAACCTCCCAGGCTCCCGAA-3′	g.30665807_30665827		0.1	
	NF1-PRS2-R	5′-AGCCCCGAGGGAATGAAAAGC-3′	g.32085390_32085410			
RT-PCR primers			NM_000267.3 (transcript variant 2)			
NF1-RT1	NF1_RT_1F	5′-GACCCTCTCCTTGCCTCTTC-3′	c.-109_-90	785	0.4	
	NF1_RT_1R	5′-TTTCTACCCAGTTCCAAAATGC-3′	c.655_676			
NF1-RT2	NF1_RT_2F	5′-AAGAAGGTTGCGCAGTTAGC-3′	c.613_632	1461	0.4	
	NF1_RT_3R	5′-CAGGGCCACTTCTAGTTTGG-3′	c.2004_2073			
NF1-RT3	NF1_RT_4F	5′-CTCTCTCCGGAAGGGAAAAG-3′	c.1968_1987	1410	0.4	
	NF1_RT_5R	5′-TGCGCACTTTCATCTTCAAC-3′	c.3358_3377			
NF1-RT4	NF1_RT_6F	5′-TGATGGAAGCCAAATCACAG-3′	c.3284_3303	1078	0.4	
	NF1_RT_31skipR	5′-GCTGCATCAAAGTTGCTTTTC-3′	c.4341_4361	1141 (variant 1)		
NF1-RT5	NF1_RT_7S	5′-AATCCTGCCATTGTCTCACC-3′	c.4180_4199	1141	0.4	
	NF1_RT_8AS	5′-CTACTAGGCAGATTTCTTCAATTTCC-3′	c.5295_5320			
NF1-RT6	NF1_RT_9F	5′-CCAGAGCACAAACCTGTGG-3′	c.5215_5237	1440	0.4	
	NF1_RT_10AS	5′-CACCTGTTGCACTGGTTTTG-3′	c.6634_6654			
NF1-RT7	NF1_RT_11F	5′-TGCTTTGACATCCTTGGAAAC-3′	c.6528_6548	1439	0.4	
	NF1_RT_12R	5′-AATTTGGATCTTGGCACAATG-3′	c.7946_7966			
NF1-RT8	NF1_RT_13F	5′-TAGCAGAGGCCAGTGTTGTG-3′	c.7865_7884	776	0.4	
	NF1_RT_13R	5′-GCAGCATTAAATTTAGGCAAGG-3′	c.*162_183			
SPRED1 long PCR primers			NC_000015.10 (chr15, GRCh38.p13)			
SPRED1_A	SPRED1_A-F	5′-ATTCCTGGAGAGGGATGGTAGAAGAGG-3′	g.38250821_38250847	19,058	0.15	
	SPRED1_A-R	5′-CCATCCAATACAGCAGTCAACACTGG-3′	g.38269853_38269878			
SPRED1_B	SPRED1_B-F	5′-TGTCTGATGTAAAAGCCACTGATGTCG-3′	g.38282586_38282612	18,830	0.15	
	SPRED1_B-R	5′-ACGTGAAACATCAGGTGTTCTGATTCC-3′	g.38301389_38301415			
SPRED1_C	SPRED1_C-F	5′-TCCTGGAATGGATCCTATACTGGAAGG-3′	g.38320922_38320948	19,080	0.15	
	SPRED1_C-R	5′-TTACTGTGTCTGGTAAAGGGCAGATGG-3′	g.38339975_38340001			
SPRED1_D	SPRED1_D-F	5′-ACCTTGCCAAAAGGACTAGACAACTGC-3′	g.38345452_38345478	17,718	0.15	
	SPRED1_D-R	5′-TAAAATAGATAGGGTTGGACGCGATGG-3′	g.38363143_38363169			

^#^ This primer set is according to the study performed by Raedt et al. [20].

**Table 2:** Summary of the clinical features of the patients.

#Patient	Age	Sex	Café au Lait Macules	Cutaneous/Subcutaneous Neurofibromas	Plexiform Neurofibroma	Axillary or Groin Freckling	Optic Pathway Glioma	Lisch Nodules	Bony Dysplasia	First Degree Relative with NF1	Other Findings	Clinical Diagnosis ^#^
NF_01 *	30	F	Y	Y	N	Y	N	Y	Y	Y	Epi, MR	Y
NF_02 *	16	F	Y	N	N	Y	N	Y	Y	N	UBO	Y
NF_03 *	12	M	Y	N	N	N	N	N	N	Y	Epi, MR	Y
NF_04 *	3	M	Y	N	N	N	N	N	N	Y	MR	Y
NF_05 *	21	F	Y	Y	Y	Y	N	Y	N	N	MR	Y
NF_06	2	M	Y	N	N	N	N	N	N	N	Epi	N
NF_07	13	M	Y	N	N	N	N	Y	N	Y	DD	Y
NF_08	8	M	Y	N	N	Y	N	N	N	N	MR, Mo	Y
NF_09	1	F	Y	N	N	N	N	N	N	N	Epi	N
NF_10	9	F	Y	Y	N	N	Y	N	N	N		Y
NF_11	14	M	Y	N	N	N	N	N	N	Y		Y
NF_12	8	M	Y	N	N	Y	N	N	N	N		Y
NF_13	1	M	Y	N	N	N	N	N	N	N		N
NF_14	9	F	Y	N	N	Y	N	N	N	N		Y
NF_15	12	M	Y	N	N	Y	N	N	N	N		Y
NF_16	5	M	Y	N	N	N	N	N	N	N	MR, UBO	N
NF_17	1	M	Y	N	N	Y	N	N	Y	N		Y
NF_18	35	F	N	Y	Y	N	N	N	N	N		Y
NF_19	4	M	Y	Y	N	Y	N	Y	N	N	MR, Epi, Mo	Y
NF_20	57	F	Y	Y	Y	Y	N	N	Y	N		Y

DD, developmental disorder; Epi, epilepsy; MR, mental retardation; Mo, quasi-Moya Moya disease; and UBO, unidentified bright object in brain MRI. * These patients were previously reported [12]. ^#^ According to a National Institutes of Health Consensus Conference in 1988 [4].

**Table 3:** Summary of the detected *NF1* variants.

#Patient	#Chrom	Pos (hg38)	dbSNP_ID	Ref	Alt	HGVS_Format (NM_000267.3)	Minor Allele Frequency	ClinVar
NF_01	chr17	31181460	rs786203448	C	T	c.625C>T p.Gln209Ter	NA	Pathogenic (known mutation)
NF_02	chr17	31229466	rs1131691122	G	T	c.2850+1G>T	NA	Pathogenic (known mutation)
NF_03	chr17	31260403	rs199474743	A	G	c.4402A>G r.4368_4402del p.Phe1457Ter	NA	Pathogenic (known mutation)
NF_06	chr17	31249093	rs137854560	C	T	c.4084C>T p.Arg1362Ter	T = 0.000008 (1/121,394, ExAC)	Pathogenic (known mutation)
NF_07	chr17	31201474	NA	A	ATT	c.1250_1251insTT p.Ile418SerfsTer56	NA	Pathogenic (SCV001571436)
NF_08	chr17	31233143	NA	CAA	C	c.3639_3640del p.Met1214AspfsTer3	NA	Pathogenic (SCV001571437)
NF_10	chr17	31226466	rs17881753	C	T	c.2033C>T p.Pro678Leu	T = 0.000298 (36/120,726, ExAC)	Benign (1); Likely benign (2); Uncertain significance (1)
NF_11	chr17	31337833	rs786203831	T	G	c.6594T>G p.Asp2198Glu	G = 0.00005 (1/21,382, ALFA Project)	Uncertain significance
NF_12	chr17	31232852	rs199474764	A	G	c.3467A>G p.Asn1156Ser	G = 0.000008 (1/121,268, ExAC)	Pathogenic (1); Uncertain significance (1)
NF_16	chr17	31200422	NA	A	T	c.889A>T p.Lys297Ter	NA	Pathogenic (SCV001571438)
NF_17	chr17	31181460	rs786203448	C	T	c.625C>T p.Gln209Ter	NA	Pathogenic (known mutation)
NF_19	chr17	31232840	rs1321848637	TACTC	T	c.3457_3460del p.Leu1153MetfsTer4	delCTCA = 0.000008 (1/125,568, TOPMED)	Pathogenic (known mutation)
NF_20	chr17	31095310	rs1060500252	A	G	c.1A>G p.Met1Val	NA	Pathogenic (known mutation)

NF_01–NF_05 were previously reported, and NF_04 and NF_05 have chromosomal-level large deletions. Novel protein truncated mutations were detected in NF_07, NF_08, and NF_16 by this study, and these mutations were submitted to ClinVar (https://www.ncbi.nlm.nih.gov/clinvar/ (accessed on 7 July 2021)) by the author (Y.N.) with the SCV numbers in the table.

**Table 4:** Summary of the in silico analysis for missense variants with unknown significance.

			PolyPhen-2					
#Patient	dbSNP_ID	SIFT	HumDiv	HumVar	Mutation Taster	Mutation Assessor	Likelihood Ratio Test (LRT)	CADD PHRED-Like Scaled C-Score (>20)
NF_10	rs17881753	Damaging (0.016)	Benign (0.124)	Possibly damaging (0.816)	Disease causing (0.999915)	Low (1.2)	Deleterious (0.000005)	21.2
NF_11	rs786203831	Tolerated (0.146)	Probably damaging (0.98)	Probably damaging (0.99)	Disease causing (0.999638)	Low (1.59)	Deleterious (0.000000)	22
NF_12	rs199474764	Damaging (0.000)	Probably damaging (0.997)	Probably damaging (1.0)	Disease causing (0.999999)	Medium (2.98)	Deleterious (0.000000)	25.6

Each cell is colour-coded depending on the degree of pathogenicity: blue for low, yellow for moderate, and red for high.

**Table 5:** Heterozygosity mapping of the *NF1* region.

Primer/#Patient	A1	C1	D2	C3	D4	C5	B2	A3	D6	B4	A5	B6	C7	A7	D8	C9	D10	C11	B8	A9	B10	D12	A11
NF_01	3/1	3/0	6/0	12/0	15/1	12/0	14/0	17/1	20/4	13/3	23/0	8/0	13/3	10/0	20/3	10/3	6/3	2/0	1/0	1/0	2/0	12/2	2/1
NF_02	0/1	2/1	1/0	0/0	0/0	2/0	3/0	1/0	11/2	10/0	1/1	20/0	8/0	3/0	15/2	7/3	7/0	25/0	7/2	7/0	15/2	19/2	2/1
NF_03	4/2	17/2	9/0	13/0	20/1	19/1	15/0	15/1	19/5	12/2	24/2	14/0	14/3	11/0	20/3	9/3	17/3	21/1	8/1	6/1	14/2	19/1	3/0
NF_04	0/0	0/0	0/0	0/0	0/0	0/0	0/0	0/0	0/0	0/0	0/0	0/0	0/0	0/0	0/0	0/0	0/0	0/0	0/0	0/0	0/0	0/0	0/0
NF_05	0/0	0/0	0/0	0/0	0/0	0/0	0/0	0/0	0/0	0/0	0/0	0/0	0/0	0/0	0/0	0/0	0/0	0/0	0/0	0/0	0/0	0/0	0/0
NF_06	0/1	1/1	2/0	0/0	1/0	3/1	0/0	0/0	1/0	0/0	0/1	1/0	0/0	1/0	1/0	0/0	1/2	0/0	2/0	0/1	0/0	0/0	0/0
NF_07	4/0	11/1	6/0	12/0	18/1	17/2	13/0	15/1	21/4	12/2	24/1	14/0	14/3	11/0	19/3	10/3	17/3	20/0	9/2	7/1	14/2	20/1	3/0
NF_08	0/0	2/0	0/0	1/0	0/0	0/0	0/0	0/1	0/0	0/0	2/0	0/0	1/0	1/0	0/0	0/1	1/0	0/0	1/0	0/0	1/1	0/0	0/0
NF_09	3/0	16/2	15/0	12/0	22/1	20/2	15/0	16/1	20/5	13/2	24/1	14/0	14/3	10/1	22/1	9/3	19/2	20/0	7/2	6/0	13/2	19/1	3/0
NF_10	3/0	5/2	5/0	12/0	18/1	15/1	14/0	16/1	19/5	13/2	30/1	10/0	14/3	10/0	18/2	9/3	14/3	21/1	7/1	6/0	13/3	18/1	2/0
NF_11	3/0	15/2	7/0	11/0	19/1	17/2	13/0	13/1	11/2	7/2	14/1	8/0	9/3	7/0	11/0	5/1	11/3	9/0	1/0	1/0	7/0	1/1	1/1
NF_12	0/0	0/1	2/0	1/0	0/0	4/1	0/0	0/0	0/0	1/0	1/0	1/0	1/0	0/0	1/0	2/0	0/0	0/0	0/0	0/0	0/0	0/0	0/0
NF_13	0/0	2/1	1/0	1/1	0/0	3/1	2/0	0/0	0/0	0/0	1/0	0/0	0/0	0/0	1/0	1/0	1/0	1/0	1/0	0/0	3/0	0/0	1/0
NF_14	0/0	1/0	0/0	0/0	0/0	2/0	1/0	1/0	0/0	0/0	0/0	0/0	0/1	1/0	1/0	0/0	3/0	0/0	0/0	0/0	0/0	0/0	0/0
NF_15	5/1	13/6	11/1	11/1	5/3	27/4	14/2	15/4	20/6	12/1	22/2	15/2	14/6	10/2	22/4	10/3	7/4	2/5	2/0	2/1	2/0	11/1	2/0
NF_16	4/0	15/2	11/0	12/0	21/1	17/1	14/0	16/1	20/5	14/1	28/1	14/0	14/3	10/0	20/3	11/3	18/3	20/1	7/1	6/1	14/2	20/1	3/0
NF_17	2/1	14/2	8/0	9/0	19/2	17/2	14/0	14/1	12/2	6/1	13/1	6/0	8/3	7/0	11/0	7/1	10/3	10/0	1/0	1/0	4/0	1/1	1/1
NF_18	2/0	8/2	11/0	11/1	18/1	17/2	12/0	13/1	11/4	6/0	6/0	3/0	7/3	7/0	11/0	5/1	7/3	9/0	1/0	1/0	3/0	1/0	1/1
NF_19	2/0	6/0	6/0	12/0	17/1	14/0	7/0	16/1	15/4	5/0	2/0	7/0	13/3	1/0	0/0	11/3	0/0	12/0	7/1	6/0	2/0	18/2	4/1
NF_20	4/1	13/2	9/0	11/0	19/1	18/2	14/0	15/1	19/0	25/6	1/0	14/0	14/3	12/0	20/2	10/4	15/3	21/0	9/3	8/0	13/1	19/1	3/0

Number of heterozygous SNV/Indel are indicated in each cell. Heterozygous regions confirmed by variants are indicated by grey, and heterozygous regions presumed by being sandwiched between two confirmed heterozygous regions are indicated by light blue. The pale red primers in the first row contain exons, and the uncoloured primers do not contain exons. NF_04 and NF_05 have large deletions of an entire allele of *NF1* (type-1 microdeletion).

## Data Availability

The raw data (fastq files) in this study were deposited in the Japanese Genotype–phenotype Archive (JGA) (https://www.ddbj.nig.ac.jp/jga/index-e.html, last (accessed on 30 May 2021)), study:JGAS000288 (https://humandbs.biosciencedbc.jp/en/hum0271-v10 (accessed on 30 May 2021)).

## Supplementary Materials

The following are available online at https://www.mdpi.com/article/10.3390/cimb43020057/s1.
