# Supplementary material for: Application of Combined Long Amplicon Sequencing (CoLAS) for Genetic Analysis of Neurofibromatosis Type 1: A Pilot Study
Source: Curr Issues Mol Biol. 2021 Jul 23;43(2):782–801. doi: 10.3390/cimb43020057 (PMC8929138; doi:10.3390/cimb43020057)
Supplement: Supplementary file 1 [file cimb-43-00057-s001.zip › cimb-1266797-supplementary.pdf]

Technical note of Combined Long Amplicon Sequencing (CoLAS) for Genetic Analysis of Neurofibromatosis type 1

I. Overview

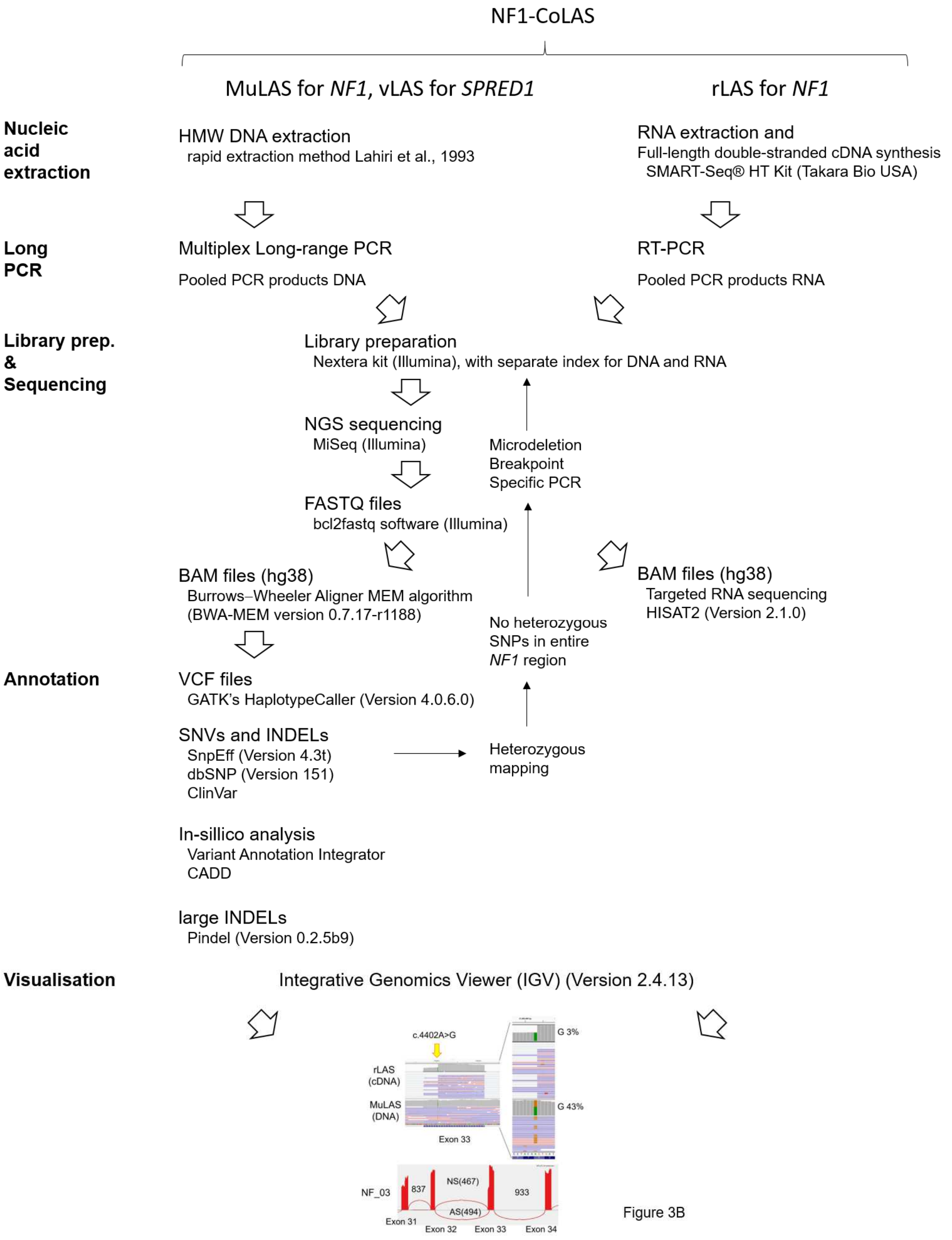

In CoLAS, DNA extraction and cDNA synthesis are performed by special methods, and the gene structure and mRNA expression can be accurately analysed by comparing the full-length gene libraries of both DNA and RNA created from Long-range PCR amplicons.

II. Technical note

1. Nucleic acid extraction

MuLAS and vLAS requires very high molecular weight DNA (average size > 50kb) to amplify long-range PCR. We strongly recommend rapid extraction method (ref.18). In our experience, conventional DNA extraction kits are not sufficient, and this method is the simplest and most inexpensive method for extracting high molecular weight DNA. To synthesize Full-length double-stranded cDNA for rLAS, we use a SMART-Seq® HT Kit (Takara Bio USA).

2. Long-range PCR

We used KOD One and KOD Multi&Epi (TOYOBO) for long-range PCR. Two disadvantages of long-range PCR are the long time required for the completion of PCR reactions and the relatively low DNA polymerase fidelity. However, these polymerases have overcome these problems by achieving rapid and high-fidelity long-range PCR amplification. According to the manufacturer's information, KOD One could be applied in extremely long-range PCR (>20 kb). KOD Multi&Epi performs uniform amplification independent of DNA sequence and length, and is capable of multiplex PCR up to 10 kb.

3. Library preparation and Sequencing

For library preparation, Nextera Flex DNA kit (illumine) or Illumina DNA Prep with Enrichment are most suitable for long PCR products. A library with very uniform coverage can be prepared in a short time, directly from PCR amplicon pool. The average length of the library is about 500 bp, sequencing is performed by Illumina MiSeq system (2 × 250 cycles).

For targeted RNA sequencing, the paired-end 250 bp reads were aligned to the reference human genome (hg38), using HISAT2 (Version 2.1.0) (ref.26). Created BAM file include splice junction sequence information, and could visualize by IGV Sashimi plot.

4. Annotation

Haplotype variant calling for each sample was performed using GATK's HaplotypeCaller (Version 4.0.6.0) (ref.22). SnpEff (Version 4.0.6.0) (ref.23) classified the deleterious impact of each variant into four classes: HIGH, MODERATE, LOW, and MODIFIER. Briefly, frameshift, nonsense, and splice acceptor/donor site mutations were classified as HIGH; missense and in-frame deletion/insertion variants were classified as MODERATE; synonymous variants were classified as LOW; and other intronic and UTR variants were classified as MODIFIER. Accordingly, HIGH call can be determined to be a protein-truncating mutation, but MODERATE cannot be immediately determined to be a non-pathogenic variant or pathogenic missense mutation. Therefore, *in-silico* analysis is required to interpret missense variants. There are much software for

in-silico analysis, but dbNFSP (ref.29) is known as one that can analyze these at once and compare the results. dbNFSP is a download programs, but it requires a large storage and is not easy to use for personal computer. Variant Annotation Integrator is a site that can analyze the main parts of dbNFSP on the web, and it is very easy to use. CADD is another type of integrated program that is also easy to use on the web (see text). It is also important that the frequency in the general population is enough low to determining whether a missense variant is pathogenic. In this study, we tentatively defined a missense variant could be pathogenic when all of the following conditions are met: frequency in general population in dbSNP is less than 0.001, majority of programs of Variant Annotation Integrator determine the variant as pathogenic, and PHRED-like scaled C-score of CADD is above 20. However, this definition is not absolute, and the final decision should be based on the results of functional assays and/or pedigree analysis.

To detect large insertion or deletion mutations, Pindel (Version 0.2.5b9) was used (Ref.17,28). We believe that this software is sufficient to detect intragenic deletions from our experience with TSC (Ref.17). Since MuLAs contain all intron sequences of NF1, by detecting heterozygous SNPs can confirm whether the copy number of each long PCR region is maintained to two. If there is no hetero SNPs in entire NF1 region, it suggests chromosomal level microdeletion, and could confirmed by breakpoint specific long PCR.

5. Visualization and Interpretation

DNA and RNA data can be visualized and compared simultaneously using IGV (Version 2.4.13) (Ref.27). This comparison allows us to confirm whether intron variants cause splicing abnormalities, or whether the expression ratio between alleles in mRNA is fluctuating compared to DNA. Also, if one mutation causes multiple splicing abnormalities, the ratio can be seen quantitatively. In this way, the integration of MuLAS and rLAS results allows for a final interpretation.
